# Exploring experiences of supports for suicide bereavement in Ireland: protocol for a national survey

**DOI:** 10.12688/hrbopenres.13437.2

**Published:** 2022-01-07

**Authors:** Selena O'Connell, Eimear Ruane-McAteer, Caroline Daly, Clíodhna O’Connor, Fiona Tuomey, Laura McDonnell, Ella Arensman, Karl Andriessen, Eve Griffin

**Affiliations:** 1 National Suicide Research Foundation, University College Cork, Cork,, Ireland; 2 School of Public Health, College of Medicine and Health, University College Cork, Cork, Ireland; 3 Healing Untold Grief Groups (HUGG), Dublin, Ireland; 4 Centre for Mental Health, Melbourne School of Population and Global Health, University of Melbourne, Parkville, VIC 3010, Australia

**Keywords:** Suicide bereavement, suicide loss, affected by suicide, help-seeking, informal support, formal support, postvention, grief.

## Abstract

Background

A suicide death impacts upon the wellbeing of close family members and friends but has also been shown to affect many people outside of this immediate circle. This will be the first large-scale national study of adults bereaved or affected by suicide in Ireland, using a cross-sectional online survey. The overarching aim will be to gain insight into the experiences of supports received by people bereaved or affected by suicide and to identify the barriers to engagement following their loss.

Methods

A cross-sectional survey will be conducted among adults in Ireland who have been bereaved or affected by suicide. This project will seek to represent people with different demographics and backgrounds in the Irish population using a multifaceted approach to survey recruitment. A range of validated measures will be used to examine participants’ current wellbeing and grief experience. A combination of closed and open-ended questions will provide participants the opportunity to share their individual experiences, the services and supports available to them, and barriers and enablers to accessing supports.

Results

Quantitative data will be analysed using descriptive statistics. Chi-squared tests will be used to compare subgroups within categorical data items, and multivariable regression models will be used to examine differences in psychosocial and physical wellbeing across key groups. Qualitative content analysis will be used for qualitative responses to open-ended questions.

Conclusions

The survey will provide an in-depth understanding of the psychosocial and mental health impacts of suicide bereavement in Ireland; insight into the range of informal and formal supports accessed; and will identify unmet needs and challenges of accessing appropriate and timely supports. The findings will inform current national actions aimed at ensuring the standardisation and quality of the services and supports for those bereaved or affected by suicide.

## Introduction

Approximately 500 suicides are recorded in Ireland annually ( Central Statistics Office, 2020). It is estimated that between six family members and up to 135 individuals may be affected by every suicide death ( Cerel *et al.*, 2019). As a consequence, more than 3,000 close family members and up to 60,000 individuals may be impacted by suicide in Ireland annually. Increasingly, researchers have begun to examine the experiences of the wider circle of people affected by a death by suicide ( Cerel *et al.*, 2019), including people who may not consider themselves bereaved by suicide but who are affected such as neighbours, passers-by, or professionals caring for the person.

It is well established that those bereaved by suicide may experience long-lasting impacts on their physical and mental health, including increased risk of suicidal thoughts and behaviours ( Spillane *et al.*, 2018), and many will require some type of support for their grief. Much of the research to-date on postvention supports (i.e., support after suicide) has focused on evaluating formal supports, particularly the positive effects of suicide-specific peer-support groups as well as for individual-based therapies ( Andriessen *et al.*, 2019; Linde *et al.*, 2017). However, an Irish study of bereaved family members reported that a range of supports, informal and formal, are required following a bereavement and that informal supports, in the form of practical and emotional support from friends and family, may be as important as formal supports ( Spillane *et al.*, 2018).

Recent research indicates that access to support remains a challenge. The Irish study by Spillane & colleagues (2018) also found that individuals’ complex needs were often not addressed by their existing support networks, with disparity in the needs and availability of supports impacting negatively on their grieving process. A survey from the United Kingdom (UK) conducted in 2010 found that 21% of those bereaved by suicide of a close relative or friend did not receive any support in their bereavement, formal or informal ( Pitman *et al.*, 2017). Focusing on the wider circle of people affected by a suicide as well as those bereaved, a recent survey in the UK found that more than 60% of study participants did not access supports following their loss ( McDonnell *et al.*, 2020). Barriers to accessing supports include perceived stigma, limited awareness of available supports by individuals and gatekeepers, financial costs, poor referral pathways and regional variation in availability ( McDonnell *et al.*, 2020; Pitman *et al.*, 2017).

In Ireland, the national strategy to reduce suicide, *Connecting for Life* (CfL; Department of Health, 2015), has instigated significant steps to identify and standardise the model of service following suicide bereavement in Ireland. This includes mapping of local statutory and non-statutory organisations offering different levels of supports and the rollout of a national suicide bereavement liaison service ( Health Service Executive, 2020). Despite the recognition of suicide bereavement as an important public health priority, and bereaved individuals identified as a suicide prevention priority group, there is a lack of in-depth studies examining their experiences of supports following bereavement ( Griffin & McMahon, 2019). There is also little evidence on factors which promote engagement, or individuals’ needs and expectations of supports.

The aim of this study is to conduct a national survey of adults bereaved or affected by suicide in Ireland to gain insight into the experiences of supports received by these individuals, and to identify the barriers and facilitators of engagement following their loss. The specific objectives will be to:

i. Examine the profile, characteristics and psychosocial wellbeing of adults bereaved or affected by suicide in Ireland.
ii. Determine the types of suicide bereavement supports - both informal and formal - utilised by adults in Ireland.
iii. Examine the experiences of receiving or engaging with supports, the associated barriers and facilitators, and the perceived gaps and unmet needs in postvention supports.

## Protocol

### Study design

A cross-sectional survey design will be employed using an online format to obtain wide representation of individuals bereaved or affected by suicide in Ireland and their experiences of supports. Quantitative and qualitative methods will be used to analyse closed and open-ended responses, respectively. A combination of methods is used to both facilitate comparison of the sample with other studies on key outcomes measures and allow for emergence of novel findings given that this area has not yet been explored on a large scale in Ireland.

### Participants

A sample of adults in Ireland (18 years and over) who have been bereaved or affected by suicide will be the target sample for this survey. This survey will seek representation across a range of demographic groups in the Irish population. We aim to represent those who have accessed services and those who haven’t, as well as those who have been under-represented in previous research including men, non-family members and work colleagues ( Pitman *et al.*, 2017). We also aim to include representation from hard-to-reach populations such as members of the Traveller community and the LGBTQI community ( Condon *et al.*, 2019; McCann & Sharek, 2014).

A broad approach will be adopted to include both individuals directly bereaved and affected by suicide ( Cerel *et al.*, 2014; McDonnell *et al.*, 2020). This is in line with the continuum of ‘suicide survivorship’ proposed by Cerel *et al.* (2014), which encompasses people exposed to suicide who are not personally affected, people affected by a suicide, and the people closest to the person, who may regularly be referred to as ‘bereaved’ and experience grief reactions on a short-term or long-term basis. In order to encourage wide participation across the aforementioned groups, examples from McDonnell *et al.* (2020) will be used throughout the survey documentation to ensure that the survey is inclusive to those directly bereaved (e.g. family and close friends) and those affected by a suicide (e.g. ” *if you knew a neighbour who has lost their partner, son or daughter, if you were the passer-by who witnessed the death or found the person, if you are front line staff who respond to an emergency ... prison officers, train drivers, health professionals responsible for their care, and other people who may have had regular social contact with the person who died ...”* ( McDonnell *et al.*, 2020; p. 59)).

### Recruitment of participants

The survey will be accessible online and a designated webpage for the survey, including a short video, will summarise the aims and scope of the project in an accessible way, providing an overview of the content of the survey and who should consider participating. Additional short videos with individuals bereaved by suicide will be used to encourage participation from a wide demographic of individuals as well as highlighting pathways to accessing supports for those who may be looking for help. 

Participants will be recruited to the survey through a combination of digital marketing and traditional approaches to maximise recruitment ( Frandsen *et al.*, 2016; Gaupp-Berghausen *et al.*, 2019). Social media advertisements will raise awareness of the survey and direct traffic to the survey webpages, primarily through Twitter, Facebook, Instagram and LinkedIn. Social media may be more helpful than traditional methods in recruiting hard-to-reach groups ( Topolovec-Vranic & Natarajan, 2016).

The main digital marketing campaign will be supplemented by traditional media advertising. Traditional media will aim to reach individuals who may not be reached by digital marketing such as older adults ( Frandsen *et al.*, 2016). Radio adverts and press releases to media outlets will highlight the survey, its purpose and call for participation.

Additional targeted approaches will be used to include the individuals who are typically under-represented in research. Recruitment will aim to reach specific individuals via organisations delivering supports and services for suicide bereavement, advocacy and patient groups, more general mental health charities, services, and gatekeeper organisations. Finally, we will also invite individuals who complete the survey to circulate within their own networks, to maximise dissemination.

### Survey and outcome measures

The survey will be distributed in an online format, via Qualtrics software (Qualtrics, Provo, UT, October 2020). The survey will gather demographic details (e.g. age, gender, employment status, area of residence, ethnicity, relationship status and living arrangements), details about the death by suicide (relationship to deceased; time since bereavement) and information on utilisation of support services following their bereavement. Some of these items follow similar wording and design to McDonnell *et al.* (2020) which will facilitate comparison of findings between the UK and Ireland.

Standardised measures are included in the survey to obtain valid and reliable measures of participants’ current wellbeing and grief experience ( Table 1). Use of these measures also facilitates comparison with other research samples. The standardised measures are the World Health Organization - Five Wellbeing Index ([WHO-5] Topp *et al.*, 2015; WHO, 1998); Patient Health Questionnaire – Anxiety and Depression Scale ([PHQ-ADS] Kroenke *et al.*, 2016); Grief Experience Questionnaire ([GEQ] Bailley *et al.*, 2000; Barrett & Scott, 1989); Personal Growth Subscale from the Hogan Grief Reaction Checklist ([HGRC] Hogan *et al.*, 2001); and the Multidimensional Scale of Perceived Social Support ([MSPSS] Zimet *et al.*, 1988). Given the length of the survey, short versions of the measures are included where they have been shown to be valid measures of the constructs ( Table 1).

**Table 1.** Standardised instruments included in survey.

Information provided	Name of original instrument and authors	Items in survey
Mental wellbeing over the past two weeks	The World Health Organization - Five Wellbeing Index ([WHO-5] Topp *et al.*, 2015; WHO, 1998)	5-item scale included
Depression and anxiety symptoms over past two weeks	Patient Health Questionnaire – Anxiety and Depression Scale ([PHQ-ADS] Kroenke *et al.*, 2016)	16-item scale included
Grief experience since bereavement	Brief version of the Grief Experience Questionnaire ([GEQ] Bailley *et al.*, 2000; Barrett & Scott, 1989)	16-item brief version included ( Feigelman *et al.*, 2009)
Personal growth following bereavement (for those bereaved more than 12 months)	Personal Growth Subscale from the Hogan Grief Reaction Checklist ([HGRC] Hogan *et al.*, 2001)	12-item Personal Growth subscale included
Perception of social support from family, friends and significant other	The Multidimensional Scale of Perceived Social Support ([MSPSS] Zimet *et al.*, 1988)	3-item version of scale included ( Slavin *et al.*, 2020)

Information on the supports accessed will be recorded using a series of questions, soliciting a combination of closed and open-ended responses. Supports will be divided into three sections, informed by the ‘Adult Bereavement Care Framework Pyramid’ ( Irish Hospice Foundation, 2020). These include general bereavement supports and suicide-specific bereavement supports grouped according to i) informal supports, ii) formal supports following bereavement that are typically community based and provide onward referral and iii) specialised supports for people bereaved or affected by suicide including counselling and peer-support groups. Participants will subsequently be asked to rate how helpful the supports were and to describe, in their own words, the ways in which the supports were/were not helpful. Open-ended questions will be used to examine participants experiences of the following: barriers and facilitators to accessing supports; availability of supports in local area; supports during the inquest process and supports for children or young people affected by the death; as well as any other thoughts they would like to share.

### Sample size and power considerations

In order to ensure that the sample will be representative of those bereaved and affected by suicide in Ireland, the profile of respondents will be monitored monthly. This will allow the research team to examine the distribution of responses in relation to key demographics. This interim monitoring, along with a flexible recruitment strategy, will allow for stratification and targeted recruitment within the general population. In order to detect associations with a prevalent factor (e.g. mean differences in depression), two groups, each with 64 respondents, would give 80% power to detect a difference in mean scores, equivalent to half a standard deviation using the standard level of statistical significance i.e. p < 0.05.

### Planned analysis

Quantitative data gathered from the survey will be analysed using descriptive statistics. Chi-squared tests will be used to examine subgroup comparisons within categorical data items, and multivariable regression models will be used to examine differences in psychosocial and physical wellbeing across key groups, with a particular focus on the relationship between social support, personal growth, and service utilisation. All statistical analyses will be completed using Stata12 IC and SPSS Version 26.

The qualitative responses to more in-depth open-ended questions will be analysed using qualitative content analysis, drawing on quotes to support the themes identified. Results of both qualitative and quantitative survey items will be analysed concurrently and used to provide a more complete picture of the experiences of survey participants ( Pluye & Hong, 2014).

### Data management and access

The data collected for this study will be anonymous. The survey will not gather any information that will directly identify a participant such as name or contact information and participants’ IP address and location will not be associated with their responses. All open-ended questions will be screened for personal details (e.g. any names, specific locations) and will be removed from the unprocessed data before proceeding. Each completed survey will be assigned an arbitrary identifier.

Nevertheless, the survey will ask participants for sensitive personal data and measures will be taken to ensure anonymity and safe storage of data in accordance with Irish Data Protection Commission and General Data Protection Regulation (GDPR). Unprocessed datafiles will not be made available or shared with anyone beyond the research team. Aggregated data will be shared with other stakeholders where appropriate. Any data which may potentially identify a participant will not be shared and the research team will consider the risk of identification when presenting data from subgroups within the survey. Given the sensitivities of the research topic we do not plan to make the microdata available via a repository. Researchers interested in accessing the data may contact the National Suicide Research Foundation ( infonsrf@ucc.ie).

Data collected for this study will be stored safely. The data files (password-protected) will be stored electronically, on a secure server, only accessible to the research team and will be accessed via encrypted devices. The data will be stored for 10 years after collection in a safe online storage facility hosted by University College Cork.

### Ethical considerations and informed consent process

This study has received ethical approval from the Clinical Research Ethics Committee of the Cork Teaching Hospital (ECM 4 (j) 10/8/2021 & ECM 3 (rr) 07/09/2021). Participants will be provided with an information leaflet detailing the study as well as access to the webpage with explanatory videos about the study before providing consent. Participants must provide consent via an opt-in tick box in order to commence the survey. It will be clear to participants that they can withdraw from the survey at any point, however, it will not be possible to retrospectively withdraw data submitted, due to the anonymous nature of the survey.

Research indicates that participation in research by those bereaved by suicide can be a positive experience for the majority, enabling altruism and personal growth ( Andriessen *et al.*, 2018). A lower proportion, 2-22%, of participants had a negative or upsetting experience, though this did not preclude it also being a helpful experience to participants. While further research is needed, time since bereavement may affect the experience of research participation ( Andriessen *et al.*, 2018). Previous data suggest that participants who are two months or greater bereaved can participate in interviews/research ( Conner *et al.*, 2012). A message on the introductory survey page will highlight that the survey could be challenging for those who have been recently bereaved and will ask people who have been recently bereaved to consider if now is a good time for them to complete the survey. Furthermore, the Personal Growth subscale from the HGRC will not be presented to those who have been bereaved for less than a year as personal growth as measured by this scale typically emerges later following bereavement ( Levi-Belz *et al.*, 2021) and these items may be construed as insensitive for those who have been recently bereaved.

Participants represent a vulnerable population and may require additional information or supports. A list of verified support services and resources along with the researchers contact details will accompany the survey homepage and will also be provided on completion. Participants who indicate a risk of harm to themselves (via one item of the PHQ-ADS or two items in the brief version of the GEQ) through their survey responses will be directed to message as to how they can access further support, a link to the verified support services and the contact details of the project team who will follow a study specific protocol based on previous research experiences and training to address participant enquiries.

### Public and Patient Involvement and Engagement (PPIE)

The research team and advisory panel for the study includes people with lived experience of suicide bereavement along with the provision of services for those bereaved or affected by suicide. These project members have provided feedback on the survey content and associated materials. Additionally, a small pool of people who have been bereaved or affected by suicide and researchers have pretested and provided feedback on the online survey. In addition, the project has appointed one independent Lived Experience Representative who will provide input across the project, including reviewing the survey tool and supporting documentation. This Representative will also contribute to the interpretation and dissemination strategy of research findings.

### Study status

This study will run for 12 months, until May 2021. The survey is due to be released in October 2021. 

## Discussion

This is the first, large-scale study of the experiences of individuals bereaved or affected by suicide in Ireland. A previous qualitative study has found that people bereaved by suicide in Ireland have difficulty accessing appropriate support ( Spillane *et al.*, 2018). The findings of this survey will provide a national profile of the needs of those who may require suicide bereavement supports and will inform service development and policy priorities for this population. The project will generate findings and knowledge which can be used by different stakeholders, aligning with several objectives of CfL. This includes the mapping of national and local supports and services across three levels of support: informal supports, formal community-based supports, and specialised supports, which correspond to the Adult Bereavement Care Pyramid developed by the Irish Hospice Foundation (2020). Additionally, the project will identify which services have been utilised most by individuals and best practice in relation to timing of supports.

This research will contribute to the existing knowledge base on the topic of suicide bereavement which has often been flagged as an under-developed area of research within suicide prevention ( Andriessen *et al.*, 2017). The data on the supports accessed in conjunction with standardised measures of wellbeing and grief will allow us to examine differences in the characteristics of people receiving supports and identify future directions for the limited literature on interventions for people bereaved by suicide ( Andriessen *et al.*, 2019). Furthermore, the survey aims to provide information on the bereavement experiences of first responders and the experience of posttraumatic growth following bereavement which are identified as important areas for research but have received limited focus so far ( Levi-Belz *et al.*, 2021; Levi-Belz, 2019; Lyra *et al.*, 2021; Maple *et al.*, 2019).

The results will provide information pertaining to the barriers experienced in accessing suicide bereavement supports and will add to the existing literature in this area, particularly from surveys in the UK ( McDonnell *et al.*, 2020; Pitman *et al.*, 2017). The findings will be beneficial to service providers in terms of proactive outreach to bereaved or affected individuals and will inform interventions and activities which seek to reduce stigma, increase public awareness, and encourage help-seeking behaviour. 

The strengths of this survey are the national scope of the survey and the use of validated measures to facilitate comparison with other studies internationally. While the multifaceted recruitment approach is a strength of the study and aims to engage hard-to-reach populations, potential limitations include underrepresentation of certain groups or individuals in areas where there are high levels of stigma associated with suicide and mental health.

### Dissemination of results

The results of this study will be published in peer-reviewed academic journals. A report detailing the survey findings will be prepared and will be available to stakeholders and members of the public. Policy briefs will be prepared and shared with key service stakeholders. The report and a summary of the findings will be available on the project webpage which will be accessible to the public. All materials detailing the results of this survey will be reviewed by a Lived Experience Representative, pending their consent.

## Data availability

No data are associated with this article.

## References

[ref-1] Andriessen K Rahman B Draper B : Prevalence of exposure to suicide: A meta-analysis of population-based studies. *J Psychiatr Res.* 2017;88:113–120. 10.1016/j.jpsychires.2017.01.017 28199930

[ref-2] Andriessen K Krysinska K Draper B : Harmful or helpful? A systematic review of how those bereaved through suicide experience research participation. *Crisis.* 2018;39(5):364–376. 10.1027/0227-5910/a000515 29618271

[ref-3] Andriessen K Krysinska K Hill NTM : Effectiveness of interventions for people bereaved through suicide: a systematic review of controlled studies of grief, psychosocial and suicide-related outcomes. *BMC Psychiatry.* 2019;19(1):49. 10.1186/s12888-019-2020-z 30700267 PMC6354344

[ref-12] Bailley SE Dunham K Kral MJ : Factor structure of the grief experience questionnaire (GEQ). *Death Stud.* 2000;24(8):721–738. 10.1080/074811800750036596 11503720

[ref-4] Barrett TW Scott TB : Development of the grief experience questionnaire. *Suicide Life Threat Behav.* 1989;19(2):201–215. 10.1111/j.1943-278x.1989.tb01033.x 2749862

[ref-6] Central Statistics Office: Briefing on Suicide Figures (provisional) - 2 ^nd^ June 2020. Dublin: The National Office for Suicide Prevention.2020. Reference Source

[ref-7] Cerel J Brown MM Maple M : How Many People Are Exposed to Suicide? Not Six. *Suicide Life Threat Behav.* 2019;49(2):529–534. 10.1111/sltb.12450 29512876

[ref-8] Cerel J McIntosh JL Neimeyer RA : The continuum of "survivorship": Definitional issues in the aftermath of suicide. *Suicide Life Threat Behav.* 2014;44(6):591–600. 10.1111/sltb.12093 24702241

[ref-9] Condon L Bedford H Ireland L : Engaging gypsy, roma, and traveller communities in research: Maximizing opportunities and overcoming challenges. *Qual Health Res.* 2019;29(9):1324–1333. 10.1177/1049732318813558 30600758 PMC7322935

[ref-10] Conner KR Beautrais AL Brent DA : The next generation of psychological autopsy studies: part 2. Interview procedures. *Suicide Life Threat Behav.* 2012;42(1):86–103. 10.1111/j.1943-278X.2011.00073.x 22288728

[ref-11] Department of Health: Connecting for Life: Ireland’s National Strategy to reduce suicide (2015–2020). Dublin: Department of Health.2015. Reference Source

[ref-13] Feigelman W Gorman BS Jordan JR : Stigmatization and suicide bereavement. *Death Stud.* 2009;33(7):591–608. 10.1080/07481180902979973 19623760

[ref-14] Frandsen M Thow M Ferguson SG : The Effectiveness Of Social Media (Facebook) Compared With More Traditional Advertising Methods for Recruiting Eligible Participants To Health Research Studies: A Randomized, Controlled Clinical Trial. *JMIR Res Protoc.* 2016;5(3):e161. 10.2196/resprot.5747 27511829 PMC4997003

[ref-15] Gaupp-Berghausen M Raser E Anaya-Boig E : Evaluation of different recruitment methods: Longitudinal, web-based, pan-european physical activity through sustainable transport approaches (PASTA) project. *J Med Internet Res.* 2019;21(5):e11492. 10.2196/11492 31066715 PMC6533046

[ref-16] Griffin E McMahon E : Suicide Bereavement Support: A Literature Review.Cork: National Suicide Research Foundation Ireland,2019. Reference Source

[ref-17] Health Service Executive: Improving suicide bereavement supports in Ireland.Dublin: Health Service Executive,2020. Reference Source

[ref-18] Hogan NS Greenfield DB Schmidt LA : Development and validation of the Hogan Grief Reaction Checklist. *Death Stud.* 2001;25(1):1–32. 10.1080/07481180125831 11503760

[ref-19] Irish Hospice Foundation: Adult bereavement care framework pyramid. A national framework.2020. Reference Source

[ref-20] Kroenke K Wu J Yu Z : Patient Health Questionnaire Anxiety and Depression Scale: Initial Validation in Three Clinical Trials. *Psychosom Med.* 2016;78(6):716–27. 10.1097/PSY.0000000000000322 27187854 PMC4927366

[ref-21] Levi-Belz Y : With a little help from my friends: A follow-up study on the contribution of interpersonal characteristics to posttraumatic growth among suicide-loss survivors. *Psychol Trauma.* 2019;11(8):895–904. 10.1037/tra0000456 30907613

[ref-22] Levi-Belz Y Krysinska K Andriessen K : "Turning personal tragedy into triumph": A systematic review and meta-analysis of studies on posttraumatic growth among suicide-loss survivors. *Psychol Trauma.* 2021;13(3):322–332. 10.1037/tra0000977 33211518

[ref-23] Linde K Treml J Steinig J : Grief interventions for people bereaved by suicide: A systematic review. *PLoS One.* 2017;12(6):e0179496. 10.1371/journal.pone.0179496 28644859 PMC5482439

[ref-24] Lyra RL McKenzie SK Every-Palmer S : Occupational exposure to suicide: A review of research on the experiences of mental health professionals and first responders. *PLoS One.* 2021;16(4):e0251038. 10.1371/journal.pone.0251038 33930087 PMC8087020

[ref-25] Maple M Poštuvan V McDonnell S : Progress in postvention: A call to a focused future to support those exposed to suicide, [Editorial]. *Crisis: The Journal of Crisis Intervention and Suicide Prevention.* 2019;40(6):379–382. 10.1027/0227-5910/a000620 31729921

[ref-26] McCann E Sharek D : Challenges to and opportunities for improving mental health services for lesbian, gay, bisexual, and transgender people in ireland: A narrative account. *Int J Ment Health Nurs.* 2014;23(6):525–533. 10.1111/inm.12081 25039498

[ref-27] McDonnell S Hunt IM Flynn S : From Grief to Hope: The Collective Voice of those Bereaved or Affected by Suicide in the UK.Manchester: University of Manchester,2020. Reference Source

[ref-28] Pitman AL Rantell K Moran P : Support received after bereavement by suicide and other sudden deaths: a cross-sectional UK study of 3432 young bereaved adults. *BMJ Open.* 2017;7(5):e014487. 10.1136/bmjopen-2016-014487 28554915 PMC5729987

[ref-29] Pluye P Hong QN : Combining the Power of Stories and the Power of Numbers: Mixed Methods Research and Mixed Studies Reviews. *Annu Rev Public Health.* 2014;35(1):29–45. 10.1146/annurev-publhealth-032013-182440 24188053

[ref-30] Slavin V Creedy DK Gamble J : Single Item Measure of Social Supports: Evaluation of construct validity during pregnancy. *J Affect Disord.* 2020;272:91–97. 10.1016/j.jad.2020.03.109 32379626

[ref-31] Spillane A Matvienko-Sikar K Larkin C : What are the physical and psychological health effects of suicide bereavement on family members? An observational and interview mixed-methods study in Ireland. *BMJ Open.* 2018;8(1):e019472. 10.1136/bmjopen-2017-019472 29331974 PMC5781012

[ref-32] Topolovec-Vranic J Natarajan K : The use of social media in recruitment for medical research studies: A scoping review. *J Med Internet Res.* 2016;18(11):e286. 10.2196/jmir.5698 27821383 PMC5118584

[ref-33] Topp CW Østergaard SD Søndergaard S : The WHO-5 Well-Being Index: A Systematic Review of the Literature. *Psychother Psychosom.* 2015;84(3):167–176. 10.1159/000376585 25831962

[ref-34] World Health Organisation: Wellbeing Measures in Primary Health Care/The Depcare Project.WHO Regional Office for Europe: Copenhagen,1998. Reference Source

[ref-36] Zimet GD Dahlem NW Zimet SG : The Multidimensional Scale of Perceived Social Support. *J Pers Assess.* 1988;52(1):30–41. 10.1207/s15327752jpa5201_2

