# Supplementary material for: Exploring experiences of supports for suicide bereavement in Ireland: protocol for a national survey
Source: HRB Open Res. 2022 Jan 7;4:114. Originally published 2021 Oct 22. [Version 2] doi: 10.12688/hrbopenres.13437.2 (PMC8602958; doi:10.12688/hrbopenres.13437.2)
Supplement: Peer review history

# Peer review: Reviewer response for version 1

**DOI:** 10.21956/hrbopenres.14641.r30798

**Published:** 2021-11-18

**Authors:** Jean Morrissey

**Affiliations:** 1 School of Nursing and Midwifery, Trinity College Dublin, The University of Dublin, Dublin, Ireland

This protocol sets out a clear and succinct overview of the proposed national survey. The rationale for undertaking this national survey is explained well. The aims of the proposed survey are very relevant and will be highly valuable to researchers, practitioners, policy makers and the public. As a qualitative researcher, I would like to have seen more information on the nature focus of the qualitative questions. Whilst I agree that one of the strengths of this survey will be the national scope of the survey, the use of so many validated measures may render this survey to be very lengthy. The latter may deter/impact recruitment.

 I wish the team well on a very worthwhile proposal.

Is the study design appropriate for the research question?

Yes

Is the rationale for, and objectives of, the study clearly described?

Yes

Are sufficient details of the methods provided to allow replication by others?

Partly

Are the datasets clearly presented in a useable and accessible format?

Not applicable

Reviewer Expertise:

Suicidology, Self-harm,  suicide bereavement

I confirm that I have read this submission and believe that I have an appropriate level of expertise to confirm that it is of an acceptable scientific standard.

# Peer review: Reviewer response for version 1

**DOI:** 10.21956/hrbopenres.14641.r30682

**Published:** 2021-11-01

**Authors:** Trisha Forbes

**Affiliations:** 1 School of Nursing and Midwifery, Medical Biology Centre, Queen's University Belfast, Belfast, UK

This protocol provides a comprehensive and thorough overview of the proposed national survey. This promises to be an exciting and hugely relevant undertaking and a vital piece of work. I am encouraged to see that there will be qualitative responses to open-ended questions included in the survey. I think that, from my perspective, it would be really interesting to explore experiences further by conducting interviews with participants, but this is clearly beyond the scope of the current study. It is definitely required that participants who identify as at risk of self-harm will be directed to resources, and I understand that it would be difficult to follow-up with them in a more proactive way, given the required level of anonymity.

 Generally, I find it hard to find fault with this protocol and with the proposed survey and wish the team all the very best with it.

Is the study design appropriate for the research question?

Yes

Is the rationale for, and objectives of, the study clearly described?

Yes

Are sufficient details of the methods provided to allow replication by others?

Yes

Are the datasets clearly presented in a useable and accessible format?

Not applicable

Reviewer Expertise:

Suicide prevention and postvention research - particularly qualitative research.

I confirm that I have read this submission and believe that I have an appropriate level of expertise to confirm that it is of an acceptable scientific standard.
